# Anthology of Dirofilariasis in Russia (1915–2017)

**DOI:** 10.3390/pathogens9040275

**Published:** 2020-04-09

**Authors:** Anatoly V. Kondrashin, Lola F. Morozova, Ekaterina V. Stepanova, Natalia A. Turbabina, Maria S. Maksimova, Evgeny N. Morozov

**Affiliations:** 1 Martsinovsky Institute of Medical Parasitology, Tropical and Vector-Borne Diseases, Sechenov University, 119435 Moscow, Russia; anakona@mail.ru (A.V.K.); lfmorozova@mail.ru (L.F.M.); stepan-kate83@mail.ru (E.V.S.); n.turbabina@mail.ru (N.A.T.); maksimovmarij@yandex.ru (M.S.M.); 2 Department of Tropical, Parasitic Diseases and Disinfectology, Russian Medical Academy of Continuous Professional Education, 125445 Moscow, Russia

**Keywords:** *Dirofilaria*, dirofilariasis, subcutaneous nodule, *D. repens*, *D. immitis*, vectors, transmission

## Abstract

Dirofilariasis is a helminths vector-borne disease caused by two species of *Dirofolaria*—*D. repens* and *D. immitis*. The former is overwhelmingly associated with human dirofilariasis. The vector of the worm are mosquitoes of the family *Culicidae* (largely *Culex*, *Aedes* and *Anopheles*). The definitive hosts of *Dirofilaria* are dogs and to a lesser extent cats. Humans are an accidental host. A total of 1200 human cases caused by *Dirofilaria* were registered in the territory of the ex-USSR during the period 1915–2016. Zonal differences have been seen in the prevalence of infected dogs and mosquitoes. Studies undertaken in the southern part of the Russian Federation (RF) revealed the prevalence of *Dirofilaria* in dogs to be 20.8% with wild variations of larva density. Studies carried out in the central part of the RF found that the prevalence of parasites in dogs was 4.1%. *Aedes* mosquitoes were infected less than *Culex* and *Anopheles* mosquitoes. The latter were infected by *D. repens* more often than *Culex* and *Aedes*. Zonal differences were also traced in regard to *Dirofilaria* prevalence in humans, thus allowing identification of three zones of risk of infection (low, moderate, and stable), reflected in a series of constructed maps. Although Dirofilariasis was known on the territory of Russia from 1915, only sporadic cases of the disease were reported occasionally. Its number was showed an increasing trend only during the 1980s–1990s, reaching the level of hundreds of cases. The majority of cases were confined to the southern parts of Russia with geographic coordinates of 43°–45° on the northern latitude. Comparison of the timing of the global trend of climate warming during the 1990s with the temporal pattern of *Dirofilaria* on the territory of Russia during the same period demonstrated a close association between two phenomena. With the continuous process of global climate warming, the incidence of dirofilariasis both in man and dogs goes unabated exemplified by the territorial expansion of the disease northwards and eastwards attaining the latitude of 56°–57° on the northern latitude in the European and Asian parts of Russia. It appears that within the period of the last 20–25 years, the population at risk has doubled. Under these circumstances, dirofilariases in Russia should be considered as an emerging public health problem necessitating the establishment of a comprehensive epidemiological monitoring system with strong entomological and veterinary components. Based on the results obtained, an appropriate control intervention could be developed.

## 1. Introduction

The first case of subcutaneous dirofilariasis was described in Palermo, Italy, in 1867 by Angelo Pace, who extracted a very thin nematode of about 100 mm in length from the upper eyelid of a nine-year-old boy. The nematode was defined as *Filaria palpebralis*. In 1898 in Sicily, Italy, Addario described the nematode from the eyelid of a woman as *Filaria conjunctivae* [1]. In Russia, the first case of dirofilariasis was detected in 1915 in a woman in a rural area in the south of the country. The parasite was extracted from the nodule located between the inner wall of eye orbit and eyeball. Parasite was defined by KI Skryabin (1917) as a new species under the name of *Loa-loa extraocularis Skrjabin* [1].

In 1940, Desportes concluded that all nematodes detected in humans and defined as *Filaria conjunctivae* belong to *Dirofilaria* genus and do not differ from *D. repens* (Railliet and Henry, 1911) [1]. In 1946, Skrjabin confirmed that all cases of subcutaneous dirofilariasis caused by nematodes under the names of *F. palpebralis*, *F. conjunctivae*, and *Loa extraocularis* were, in fact, due to *D. repens*. It was established that *D. repens* is a common parasite in dogs in Europe and Asia, and to a lesser extent, in Africa and in the Americas [2].

Studies on the development of *D. repens* in *Aedes fasciata* mosquitoes were initiated by P. Bernard and G. Bauche in 1913 [1].

The definitive hosts of *D. repens* and *D. immitis* are animals of the families *Canidae*, *Felidae*, and *Viverridae*. Dogs are the most important source for human transmission, their infectivity in Russia varies within a broad range 1.4–44.5% [1]. Russia has been one of the most important endemic regions worldwide for *D. repens*, the most prevalent *Dirofilaria* species in Russia. The geographic area of dirofilariasis is determined by the presence of definite host and vector(s). Therefore, outside temperature is of great importance, allowing for the development of *Dirofilaria* into the infective stage (L3) in the mosquito [3]. Exponential increase of dirofilariais in man and in dogs reported during the last 20–30 years in Russia dictates that the national authorities should consider the situation as an emerging public health problem necessitating the implementation of efficacious preventive and control measures.

## 2. Epidemiology of Dirofilariasis in the Ex-USSR

In the absence of complete official data, only information from various sources (mainly publications in Russian and through PubMed, as well as from the reports of health treatment facilities) were analyzed in our review. A total of 1200 cases caused by *D. repens* were registered in the territory of the ex-USSR during the period 1915–2016 [1,4,5,6,7,8]. The dynamics of *D. repens* incidence during this period is presented in Table 1.

It can be seen that there was a well-established trend of an increased number of cases starting from 1999 onwards. A probable explanation for this phenomenon is the effect of climate warming starting from the 1970s, facilitating the efficiency of the transmission by mosquitoes [9,10,11].

### 2.1. Age-Wise and Sex-Wise of Cases

There was no marked preponderance of cases in regard to age. All age groups were affected, with some peak of prevalence among 30–39 and 50–59-year-olds. The average age of infected persons was shown to be 38 years; the youngest case was in a 3-year-old and the oldest was more than 70 years old [6,9,12]. There was no difference in regard to the rates of infected women and men.

### 2.2. Seasonality of Human Dirofilariasis

There were two seasonal peaks of cases. One peak was in the month of January and the second in June. The seasonality of invasion was well defined during the warm months—from June through September. As a rule, more than 50% of all cases were reported during this period. Consequently, maximal microfilaria density in dogs was reported in the third quarter of the calendar year [4,9,13,14].

### 2.3. Geographical Confinement of Human Dirofilariasis

At the beginning of the 2000s, there were around 800 cases of *D. repens* recorded in the world, of which 267 cases were in the Russian Federation, 100 cases in the other republics of the ex-USSR, 178 cases in Italy, and 250 cases elsewhere in the world [9]. 

Prior to the disintegration of the USSR (1991–1993), the majority of cases were detected in the territory of the Russian Federation (62%) and Ukraine (21%). The rest of the cases (17%) were registered in Kazakhstan (5%), Uzbekistan (4%), Georgia (4%), and Turkmenistan (4%) [9]. 

During the period 1960–1996, the northern border area of the occurrence of indigenous cases did not cross the line of 53°–54° on the northern latitude. The bulk of the cases in the territory of the Russian Federation were registered in the Krasnodar region, and in the cities of Astrahan, Volgograd, and Saratov [6]. A series of sporadic indigenous cases were reported in other cities and territories of Russia—Khabarovsk [15], Nishni Novgorod [16], Tula [17], Amursk [18], Magnitogorsk [19], Volgograd [20], Ulyanovsk [21], Novosibirsk [22], Tiumen [23], Barnaul [24], Vladivostok [25], and Rostov [26].

In Ukraine, dirofilariasis cases were registered in southern regions [27]. On the whole, the territories with reported indigenous cases were found within the limits of the isotherm of the month of January with temperatures from 0 to +16 °C, and the isotherm of July with temperatures from +16 to >32 °C and with fluctuations of annual cumulative sun radiation of 80–160 kcal/cm [1].

Apart from the autochthon cases, there were cases that might have been contracted outside of the permanent residence of the patient, particularly while visiting foreign countries with dirofilaria transmission [28]. Contraction of infection could also occur in the case of travel of hosts with their pets into *Dirofilaria*-prone areas [29]. For instance, contraction of infection by service dogs in Chechen Republic resulted, on their return to the Kirov region, in the establishment of indigenous transmission among local residents [30].

### 2.4. Detection and Diagnosis of Human Dirofilariasis Cases 

Patients themselves actively sought medical assistance from various health treatment facilities and among different medical specialists such as ophthalmologists, surgeons, oncologists, dermatologists, venereal disease specialists, dentists, urologists, and others. Surgical interventions were required in all cases, as all attempts to treat the disease with drugs did not produce the expected results [1].

In the majority of cases, the primary diagnosis of dirofilariasis was a fibroma, atheroma, cyst, and/or nodule/tumor. If parasites were not removed surgically and in a timely manner, the nodule would convert into an abscess containing helminths. In the opened abscess, the parasite could be found encapsulated. 

Cases were described when the patient himself recovered helminths by scratching an itchy surface of a subcutaneous nodule. The parasitological diagnosis of the infection was based on histological and/or morphological identification of the helminths from the filarial nodule. It appeared that morphological identification was more difficult in the case of dead parasites [31,32].

Morphological examination of parasites included species identification, sex, size, and the degree of sexual maturity, along with additional examination of the content of its uterus and vagina in respect of the presence of microfilaria [33,34,35]. Regarding the latter, consensus did not exist among the researchers on a putative development of the *Dirofilaria* female attaining a gravid stage in the presence of male(s) in the organism of human beings. Since long ago, infected humans were considered a “biological dead end” for dirofilariae. Their migration in the body was defined as “cutaneous larva migrans”. However, this notion did not commensurate with the results of studies in Russia in regard to the size of the worms in the body of infected humans. Based on the *D. repens* surgically recovered from 140 infected patients, it was demonstrated that 51.4% of the specimens were more than 120 mm long. Thus, the conclusion was that these worms could be considered adult puberty females [36]. 

Similar results were obtained in another study in the Rostov region of Russia, involving 266 dirofilariasis patients, where the proportion of mature female and adult male worms were found to be 10.5% and 0.9%, respectively [6]. Thus, the obtained data supported the view that *Dirofilaria* could develop and attain a mature stage in a human host [5,37]. 

In the process of routine epidemiological and clinical examination of one dirofilariasis patient, a resident of Tollyatti city, Supriaga [33], it was found that the microfilariae of *D. repens* was presented in the subcutaneous punctate. This was the first case in Russia, proving that a single *D. repens* female can achieve sexual maturity in the human body and can become gravid in the presence of even a single male specimen [33]. Later, similar results were obtained in Italy. A case was reported in which microfilariae were detected in the fine-needled aspirate of a *Dirofilaria* subcutaneous node [38]. It was confirmed that complete development and fertilization of *D. repens* worms in human hosts might occur [38]. In the following years, microfilariae were detected in the blood of a patient [5,39,40], such as in Europe, where 10 cases of *D. repens* were found in the lung; two similar cases were described in Russia as well [41]. 

Based on the above evidence, it was argued that it would be inaccurate to relate human dirofilariasis to the group of helminthic diseases defined as “cutaneous larva migrans”. It seems that it is inconsistent with the biology of a causative agent accomplishing migration at the adult stage in the host’s organism, and it does not direct a physician to establish correct clinical and parasitological diagnosis [33]. Therefore, it was proposed to use a new definition, “*imago migrans*”, and to consider humans a facultative host of the worm [5]. 

Migration of adult worms—“*imago migrans*”—is a very important symptom in the diagnosis of dirofilariasis, indicating the achievement of sexual maturity by the female. Genetically determined, the female becomes very active in searching for a mature male for mating [36]. Finding encapsulated females [6] might indicate that they have attained sexual maturity, but without becoming gravid in the absence of a male, they lose their activity and enter a “sleeping” stage. The organism of a human, by using its immunological preventive factors, manages to encapsulate parasites, irrespective of their localization [6]. Nevertheless, even when encapsulated, *Dirofilaria* females might be alive and become active after a long period of time [5]. Therefore, it may be assumed that migration of the adult *Dirofilaria* female is determined genetically and that this ability was evolutionary acquired in the same manner as in the case of dracunculiasis, loa loa, and others [5]. 

In the Russian Federation, like in other countries, the use of PCR in conjunction with clinical and epidemiological investigations of the patient constitutes the basis of dirofilariasis diagnosis [42]. This approach was initiated in Russia at the beginning of the 2000s. In the course of investigations, Russian researchers modified the PCR by choosing and synthesizing primers and selecting amplification regimens for them. Agarose gel bands were obtained that corresponded to a PCR fragment length nucleotide sequence equal to 245 bp for *D. repens* and 656 bp for *D. immitis*. There was a 100% agreement between the results of the PCR and the microscopic examination of sera from 32 dogs and one female patient with low parasitemia [32].

### 2.5. Localization of Dirofilaria

There were great variations in regard to localization of the worm in the body of the patient. The head, face, and eyes were more frequently affected by the parasite than any other part of the body. The reason is that a patient would notice the signs of infection much earlier compared with other locations.

In the majority of cases, the parasite was located under the skin and mucous membrane. In almost 50% of the total cases, the parasite was found under the skin of the eyelids, conjunctive, and inside the eyeball [29,43]. Considerable numbers of *Dirofilaria* nodules were found under the skin of the extremities and on the trunk. Filarial nodules were not at all rare in women’s breasts (around 10% of total cases) [44,45]. In a few sporadic cases, dirofilariae were identified in men’s genitalia [12] and under the mucous membranes of the mouth and pharynx. A very rare case of funiculitis of the right scrotal spermatic cord, caused by *D. repens*, was described in a 12-year-old boy from the Tambov region of Russia [34]. 

### 2.6. Clinical Manifestations of Human Dirofilariasis 

The period between contraction of infection and the formation of filarial nodule was variable and could be somewhere between 1 month and 2 years. The incubation period varied from 3 months to 12 months among 11 patients detected in the Moscow region during the period March 2001–August 2002 [29]. The first manifestations of the disease—initially a painless tumor/nodule—were accompanied by itching and burning of various degrees [46,47,48].

Patients sometimes complained of an almost immediate appearance of a strong itching in the site of a mosquito bite. Subcutaneous migration of the parasite was the most remarkable symptom of the infection in the majority of cases. The distance covered by the helminth could be up to 10–15 cm during a 24-hour period. In certain cases, migration of the parasite was provoked by the use of physiotherapy procedures, rubbing of ointment and the like. A specific sign of dirofilariasis was a patient’s uneasy feeling that something is crawling inside the nodule. Cases of repeated infection were not at all rare [49].

Among the other reported symptoms of infection were headaches, malaise, vomiting, fever, and pain in the site of dirofilaria-caused nodule with irradiation along the course of nerve [46,50,51,52]. Eosinophilia is not typical for the disease, although it was observed in 8–11% of patients [2]. One of the rare manifestations of human dirofilariasis was erysipelatous inflammation of the outer side of the right leg in a 68-year-old woman in the Moscow region [34].

In the overwhelming majority of cases, only a single specimen of the parasite was discovered—as a rule, a non-gravid female up to 320 mm in length. There was a single case of discovery of two males of *D. repens* in one capsule [53]. 

### 2.7. Dirofilariasis in Dogs 

A total of 1824 dogs were examined regarding the prevalence of dirofilariasis in the Rostov region of the Russian Federation during 1997–2007. The region is known for its steady local transmission of *Dirofilaria* during a number of years [6]. The purpose of the studies was to carry out comparative analysis of the clinical and biochemical parameters of the blood among infected and healthy dogs [30]. *Dirofilaria* species were identified based on morphological differences between *D. repens* and *D. immitis*. Biochemical examination was done by using commercial test-systems, and PCR methods were used in the examination of the dogs’ blood. The protein fractions of the blood were examined by the use of disk electrophoresis [30]. 

The results of the studies revealed that the prevalence of dirofilariasis in dogs was 20.8%, with the density of infection being from 10–20 up to 5000 larva/mL of blood. One of the most remarkable characteristics was the almost exponential increase in prevalence of *Dirofilaria* in dogs from 8.0% in 1997 to 31.5% in 2005. For confirmation of the results of morphological species identification, 40 samples of blood from infected dogs were examined by PCR, the results of which demonstrated the presence of *D. repens* (40.0%), *D. immitis* (27.5%), and mixed infection by both species (32.5%) [54]. 

There was a seasonal fluctuation of *Dirofilaria* prevalence, with some preponderance in summer (27.5%) and winter (21.2%) as compared to the spring (19.5%) and autumn months (18.6%). The incubation period varied from 6 to 9 months for both species [54].

A particularly high prevalence of *D. repens* infection was observed in the Rostov region in dogs employed in the police and armed forces. It was demonstrated that these dogs might pose a particular problem, since they serve as epidemiologically important amplifiers within the region [3].

Large-scale studies on the prevalence of *Dirofilaria* among dogs were carried out in the Moscow region in 2003–2007, where local transmission of dirofilariasis took place on a smaller scale (39 human cases up to 2007) compared to that in the Rostov region described above. A total of 3371 dogs were examined regarding the presence of microfilaria in their blood. The identification of *D. immitis* was done by examination of excretory and anus pores, and *D. repens* by anus pores only. Both Romanovsky–Giemsa staining and native smear were used. A modified PCR test was used for isolation of *Dirofilaria* DNA in local *Aedes*, *Anopheles*, and *Culex* mosquitoes [32].

The results of the studies showed that the prevalence of the parasite in dogs was 4.1%. The density of *Dirofilaria* in the blood varied from 2 to 3650 microfilaria/mL. *D. immitis* was overwhelmingly prevalent (82.7%), followed by *D. repens* (17.3%). Mixed cases were found in eight dogs. The severity of filariasis in dogs was related to the location of the parasite in the body of the animal. In the case of *D. immitis* being located in the heart and lungs, in 32.5% of total cases, the manifestations of the disease were sub-clinical, or even without any symptoms, and diagnosis was done only on the basis of finding the microfilaria in the blood. A severe form of the disease, caused by *D. immitis* was observed in 37.7% of the infected dogs. 

The subcutaneous form of the disease caused by *D. repens* was found in nine dogs with moderate clinical manifestations. Dogs infected with both forms of *Dirofilaria* experienced severe clinical manifestations of the disease [54]. 

### 2.8. Dirofilaria in Vectors

Due to differences in mosquito ecology in the territory of the Russian Federation, several local proved vectors of *Dirofilaria* might support a “relay” transmission of infection. This occurs when one mosquito species starts transmission and another species takes over, thus prolonging the season of transmission. 

The first generation of *Anopheles* mosquito species appears after hibernation in the second half of May, in parallel with the fraction of *Aedes* mosquitoes capable of breeding in cool waters. The density of both species is not high during the month of May, but increases steadily in the month of June, at the expense of breeding of the thermophile *Aedes* species. Later, *Culex* mosquitoes join and the maximal density is recorded in the months of July–August for all three species. 

Studies on the prevalence of infected mosquitoes were carried out in the territory of the Rostov region, known for its *Dirofilaria* endemicity (total of 169 cases during 2000–2007) [30]. Observations were also extended to the neighboring towns of Tuapse and Maikop [54]. *Aedes* mosquitoes were infected almost twice as much as *Culex* and several times higher than *Anopheles* mosquitoes (18.2%, 10.8%, and 1.3%, respectively). These data are in agreement with the prevalence of *Dirofilaria* in dogs, which, in 2005–2006, varied from 17.0% in Tuapse to 25.6% in Maikop [54]. 

Rakova (2012) carried out studies on the prevalence of infected mosquitoes, employing the PCR technique in the Moscow, Nizhni Novgorod, and Tver’ regions in the central part of the European territory of Russia from 2006. It was found that the prevalence of *D. repens* was 1.33%, and *D. immitis* was 0.52% [55]. Seasonal variations were noticed as well, with peaks in August–September of 12.0%. Unlike in the Rostov region, *Aedes* mosquitoes were infected less than *Culex* and *Anopheles* mosquitoes (3.59%, 3.72%, and 2.64%, respectively). *Anopheles* mosquitoes were infected with *D. repens* more frequently than *Culex* and *Aedes* mosquitoes (3.35%, 2.31%, and 1.86%, respectively) [56].

Entomological studies were carried out in the Tula region (190 km south-east of Moscow) during 2013–2014 using PCR [55,57]. *D. repens* and *D. immitis* were found in a total of 12 species of mosquitoes, with a total prevalence of 2.5%. The majority of the infected mosquitoes were found in the *Aedes* (3.5%) and in *Culex* (1.8%) species [56].

There was an appreciable difference of *Dirofilaria* infectivity in various species of mosquitoes within the genera of *Aedes*, *Culex*, and *Anopheles* in the territory of Russia. The highest *Dirofilaria* infectivity was registered in *Aedes caspius* (81.8%), *Culex pipiens fatigans* (14.2%), and *Anopheles hyrcanus* (70%) [57,58].

### 2.9. Epidemiological Classification of Human Dirofilariasis Cases

**Figure 1:**
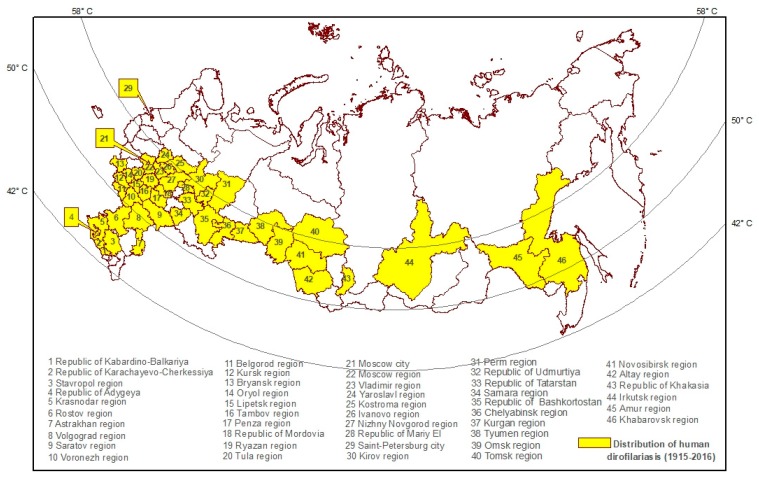
Distribution of human dirofilariasis, Russia, 1915–2016.

The majority of human dirofilariasis cases were found in towns and cities of the country (Figure 1). However, not all of these cases represent local transmission of the disease. Epidemiological investigation of the cases revealed that quite often, infected persons had, in their anamnesis, a period of time, particularly during summer and autumn, that they spent in rural areas endemic for dirofilariasis [57]. Similarly, in many instances, infection could be contracted abroad in countries with well-known foci of dirofilariasis [57].

Comparative studies on the prevalence of *Dirofilaria* in dogs in the Republic of Kalmykia (Southern Russia) revealed that *D. repens* was predominant both in rural and urban areas (23.6% and 15.5%, respectively). *D. immitis* was found only in a few dogs [59]. The highest prevalence of *D. repens* was noted in 4–6-year-old dogs (33.3%); puppies were not infected at all. The density of microfilaria in 20 mL of blood in rural dogs was 247.3 compared to 112.7 in urban dogs.

The relative preponderance of dirofilariasis in rural dogs was explained by the highest density of mosquitoes and by closer contact with local people in rural areas as compared to urban population [60].

## 3. Control and Prevention of Dirofilariasis in the Russian Federation

At present, in the absence of efficient specific medicaments for the treatment of human dirofilariasis, its prevention is rested with the control of vectors and the treatment of dogs [60].

Experience of treating dogs with *Dironet* (a new combination drug adopted in Russia, consisting of “pirantel pamoat”, “praziquantel”, and “ivermectin”) was obtained by treating 345 service dogs in Astrakhan region, Russia in 2008 [61]. Prior to the administration of drugs, the prevalence of *D. immitis* in service dogs was 3.5% compared to 20.8% in dogs in the private sector. *Dironet* tablets were given monthly (one tablet per 10 kg weight) during the transmission season (April through October). The efficacy of the treatment was 100% [61]; however, whether such an approach is feasible on a large scale is still an open issue.

Therefore, the main task of health authorities in regard to human dirofilariasis lies in the organization of measures aimed at the prevention of clinical complications of the disease. Importantly, health authorities should be well aware of the pattern of distribution of the infection in their territories and should be conversant with the methods of detection, treatment, and prophylaxis. To prevent infective mosquito bites, there is a need to use anti-vector measures. There is also a need to obligatory record and report all cases of *D. repens* infection and to carry out epidemiological investigation and classification of every case. 

To facilitate the health authorities to assess the risk of dirofilariasis in the territory of the Russian Federation, three eco-epidemiological zones were identified: low, moderate, and stable [4,62]. This was done on the basis of the geographical distribution of 564 cases of infection caused by *D. repens* during 1915–2008. The basic characteristics of each identified zone are presented in Table 2.

It was found that the climatic conditions in all three zones were favorable for the achievement of an infectious stage of *D. repens* in local mosquitoes. Indigenous transmission of filariasis may take place in the territories to the south of the latitude of 58° north in the European part of Russia and in western Siberia, and to the south of the latitude of 50° north in the far east of Russia. Borderline in the south is 41° north in the European part and 42° north in the far east [4].

Furthermore, a simplified approach for the construction of a map on the risk of dirofilariais in the territory of the Russian Federation was deployed by Morozova et al [64]. This approach took into account that *Dirofilaria* transmission depends upon successful larvae development in a mosquito, which, in turn, requires favorable environmental conditions, particularly a certain sum of warm temperatures. Suitable climate conditions for a single infection development cycle of *Dirofilaria* can be defined by the following equation:

**Σ [T °C average actual − T °C threshold (14 °C)] > 130° degree-days**, where:

- T °C average actual is an average daily air temperature;
- T °C (14 °C) is the lowest temperature threshold below which *Dirofilaria* development within a mosquito vector does not occur;
- 130 degrees/days is “a sum of effective temperatures” (SET) or “growing degree day” (adopted in several models in the West) needed for *Dirofilaria* larvae to reach infectivity. The concept of SET was originally developed and successfully applied in Russia in the control of malaria [65]. This approach was applied to other vector-borne diseases as well, for example, for cutaneous leishmaniasis [66].

To construct a map, long-term average air temperature data for the month of July were obtained from the Meteorological Office of Russia for the period 1937–2016. July is the warmest month of the year, with the highest density of vectors and microfilariae in the blood of infected dogs. An isotherm of +14 °C for July was chosen as the northern border of a potential dirofilariasis endemic area in the territory of the Russian Federation. 

It appears that the potential endemic regions based on the SET data generally match the distribution of registered human cases of dirofilariasis [4]. On the other hand, the SET was not sufficient to support local transmission of *Dirofilaria* in the cities of Anadyr, Magadan, Murmansk, Naryan-Mar, and Salekhard, as well as to the north of these cities.

**Figure 2:**
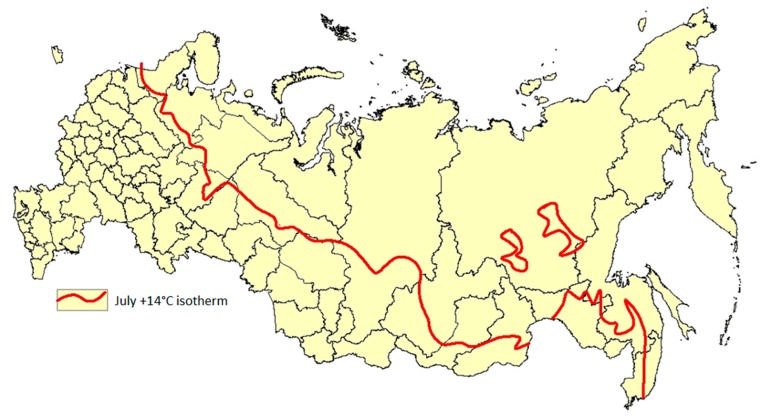
The theoretical northern border of the dirofilariasis range in the the Russian Federation.

The predictive northern border of the area of dirofilariasis in the territory of the Russian Federation is shown in Figure 2. 

It should be kept in mind that the parameters of a long-term annual average air temperature may serve only as guidance criteria. They cannot reliably characterize the conditions for a given year. 

To verify a practical utility of the July isotherm of +14 °C, the number of possible successive cycles (“turnovers”) of *Dirofilaria* larvae development in a mosquito vector was calculated in 19 administrative territories of Russia over a 10-year period. Variation of this parameter was observed across all territories. However, the range of these variations was different. The smallest range was observed in the north-western region (1–3 turnovers) and Kaliningrad Region (3–5). The largest ranges were observed in Oryol Region (3–7), Tula Region (4–9), and Belgorod Region (7–10). 

It was noted that local transmission is possible from introduced infected animals in certain years with unusually warm weather, even under normally unfavorable conditions, in territories like the Republic of Karelia, Arkhangelsk and Murmansk Region, and the Nenets Autonomous Okrug (district), which may result in sporadic local cases of dirofilariasis among dogs and humans. Indeed, such occasional cases have been reported in the Republic of Karelia and Murmansk Region. 

## 4. Conclusions

Analysis of the temporal and spatial distribution of human dirofilariasis in the territory of the former USSR and the present Russian Federation revealed that infection is well established in various parts of the latter. There is an urgent need to develop efficient medicaments for the efficacious treatment cases of human dirofilariasis, as, at present, all available drugs are not sufficiently effective. Moreover, the available methods to prevent the disease through the treatment of dogs and the control of vectors are very costly and difficult to carry out on a large scale. There is a need to obligatorily record and report all cases of *D. repens* in humans and to carry out an epidemiological investigation and classification of cases.

Experience demonstrates the necessity of establishing a permanent epidemiological monitoring system with strong veterinary and entomology components enabling the monitoring of the territories under risk of local transmission of *Dirofilaria* infection. The results of entomological observations would facilitate epidemiological investigations and classification of cases and serve as a basis for implementation of preventive and control activities. The latter should include collection, mosquito species identification, and infectivity of vectors. The value of the latter should be obtained through the use of modern molecular and biological methods in general, and PCR-modified tests in particular. It is proposed to carry out studies on the structure of transmission season of *D. repens*, depending on ambient temperature in regard to (a) the beginning of the transmission season, (b) its completion, and (c) the number of turnover(s) of infection. Finally, the limitation of the paper is seen in the absence of complete information on dirofilariasis in Russia from official sources. Therefore, it can be presumed that actual incidence of the disease might be higher than presented in this paper.

## Figures and Tables

**Table 1:** Cases of human dirofilariasis in the ex-USSR (1915–2016).

1915–1944	1945–1954	1955–1964	1965–1974	1975–1980	1981–1985	1986–1990	1991–1995	1996–1998 *	1999–2001 *	2002–2008 *	2008–2016 *
9	5	12	21	13	25	13	21	28	114	303	636

* The Russian Federation only.

**Table 2:** Comparative data on the zonal distribution of *D. repens* in Russia.

	Zone	Host		Vectors (Infected)			Reference
		Definitive—Dog (%) (Prevalence)	Facultative—Human (Number of Patients)	*Aedes* (%)	*Culex* (%)	*Anopheles* (%)	
1	Low	7.7 (service)	64	2.6	3.6	3.7	Rakova, 2013 [63]
2	Moderate	7.3 (service)	36	4.8	6.2	<1.0	Ivanova, 2013 [15]
		4.2 (stray)					
3	Stable	25.6	244	18.2	10.8	1.3	Nagornyi et al., 2012 [62]

## Data Availability

All documents and publications in Russian are available from the Archive and Library at the Martsinovski Institute of the Sechenov University, Moscow, Russian Federation.

## Abbreviations

PCR	Polymerase Chain Reaction
USSR	Union of Soviet Socialist Republics
WHO	World Health Organization
